# Streamlining OneDep Depositions of Multiple Related 3DEM Entries with pdb_extract

**DOI:** 10.1063/4.0000932

**Published:** 2025-10-27

**Authors:** Justin W. Flatt, Chenghua Shao, Brian P. Hudson, Irina Persikova, Yuhe Liang, Zukang Feng, Ezra Peisach, Jasmine Young, wwPDB OneDep Team, Stephen K. Burley

**Affiliations:** 1 RCSB Protein Data Bank, Rutgers, The State University of New Jersey, Piscataway, NJ, USA; 2 PDBe, EMBL-European Bioinformatics Institute, Hinxton, Cambridgeshire CB10 1SD, United Kingdom; 3 Institute for Protein Research, Osaka University, Suita, Osaka, Japan; 4 EMDB, EMBL- European Bioinformatics Institute, Hinxton, Cambridgeshire CB10 1SD, United Kingdom; 5 BMRB, UConn Health, Farmington, CT, USA; 6 RCSB Protein Data Bank, San Diego Supercomputer Center, University of California San Diego, San Diego, CA, USA

## Abstract

The Protein Data Bank (PDB) was established in 1971 as the first open-access digital resource in biology with just seven deposited protein structures. Today, the single global PDB archive houses more than 233,000 experimentally determined three-dimensional (3D) structures of biological macromolecules, covering a vast array of molecular components and assemblies found in biological systems. Open access to PDB data without limitations on usage allows researchers around the world to explore 3D structures for insights into disease mechanisms, therapeutic targets, and molecular interactions. Open access to PDB data has also played a critical role in the cryo-EM resolution revolution, as well as the development of AI/ML software tools (AlphaFold, RosettaFold, OpenFold, *etc*.). Today, more than one million public-domain computed structure models are being delivered alongside experimentally determined PDB structures by the Research Collaboratory for Structural Bioinformatics Protein Data Bank (RCSB PDB, RCSB.org) [1-2]. RCSB PDB is a founding member of the Worldwide Protein Data Bank (wwPDB) partnership (www.pdb.org), which collaboratively manages three wwPDB Core Archives: PDB for atomic coordinates and macromolecular crystallography data; EMDB for 3DEM density maps; and BMRB for NMR data. As the US data center for the wwPDB, RCSB PDB oversees the deposition, validation, and biocuration of newly determined biostructures, including all 3DEM data, across the Americas and Oceania.

Cryo-EM is still rapidly evolving, with AI and automation poised to help accelerate the data revolution, which will enable the field to determine more than 100,000 3DEM structures over the next five years. During this time, cryo-EM will surpass X-ray crystallography as the dominant method in structural biology [3-5]. As 3DEM depositions to PDB and EMDB grow in complexity and number, data submission process will become increasingly burdensome. For example, a single map-model composite entry may require five or more supporting depositions to include all focused and consensus maps, per wwPDB policies. These requirements create a significant challenge when a depositor submits multiple composite entries, each requiring additional depositions, leading to batch sizes of tens or even hundreds of depositions. To better support the growing needs of the 3DEM community, pdb_extract (https://pdb-extract.wwpdb.org/) has been enhanced to parse metadata and streamline the preparation of EM-derived coordinates and reusable metadata template files for map-only entries submitted to OneDep [6-7]. This presentation will focus on how the tool simplifies the deposition process, significantly reducing the manual input required at the depUI and easing the submission of large numbers of multiple related entries.
